# Association between Small Fetuses and Puberty Timing: A Systematic Review and Meta-Analysis

**DOI:** 10.3390/ijerph14111377

**Published:** 2017-11-13

**Authors:** Xu Deng, Wenyan Li, Yan Luo, Shudan Liu, Yi Wen, Qin Liu

**Affiliations:** School of Public Health and Management, Research Center for Medicine and Social Development, Collaborative Innovation Center of Social Risks Governance in Health, Chongqing Medical University, Chongqing 400016, China; dengxuroy@163.com (X.D.); lwy562011@163.com (W.L.); ly987146114@163.com (Y.L.); liushudankelly@163.com (S.L.); wen77820043@163.com (Y.W.)

**Keywords:** small fetuses, small for gestational age, low birth weight, puberty, systematic review, meta-analysis

## Abstract

*Background*: Epidemiological studies reporting the effect of small fetuses (SF) on puberty development have shown inconsistent results. *Objective*: To examine current study evidence and determine the strength and direction of the association between SF and puberty timing. *Methods*: PubMed, OVID, Web of Science, EBSCO, and four Chinese databases were searched from their date of inception to February 2016. All cohort studies that examined the association between SF and puberty timing in children were identified. Two reviewers independently screened the studies, assessed the quality of included studies, and extracted the data. The quality of the included cohort studies was assessed by the Newcastle–Ottawa Scale. Risk ratio (RR), Weighted Mean Difference (WMD), and 95% confidence intervals (CIs) were calculated and pooled by RevMan5.3 (Cochrane Collaboration, London, UK). *Results*: A total of 10 cohort studies involving 2366 subjects was included in the final analysis. The pooled estimates showed that SF did not significantly increase the number of pubertal children in boys (RR: 0.97; 95% CI: 0.82 to 1.15), or in girls (RR: 0.91; 95% CI: 0.79 to 1.04). Compared with the control group, the SF group had an earlier onset of puberty in girls (WMD: −0.64; 95% CI: −1.21 to −0.06), and in precocious pubarche (PP) girls (WMD: −0.10; 95% CI: −0.13 to −0.07). There was no difference in the onset of puberty in boys (WMD: −0.48; 95% CI: −1.45 to 0.50) between SF and control groups. The pooled result indicated an earlier age at menarche in girls born small for gestational age (WMD: −0.30; 95% CI: −0.58 to −0.03), but no difference in the age at menarche in the SF group of PP girls. *Conclusions*: SF may be associated with an earlier age of onset of puberty, especially among girls, as well as earlier age at menarche for girls. Well-designed studies with larger sample sizes and long-term follow-up among different countries and ethnicities are needed.

## 1. Introduction

There has been growing concern about the secular trend of early puberty timing in children, especially in girls [1]. In Europe, the age of menarche has declined significantly since the 19th century [2,3], as it has for American girls in the 20th century [4]. After a stabilization of menarcheal age for 3–4 decades, some data suggest that a downward trend maybe occurring [5,6]. Early puberty may cause psychosocial and other health problems in children by compromising growth, and increasing risk for behavioral disorders, early onset of sexual activity and potential abuse, and submitting the child to inadequate conditions [7]. People with precocious puberty (PP) which is defined as onset of puberty before 8 years in girls or 9 years in boys can be considered a special subgroup of early puberty [8]. 

It is well documented that the causes of early puberty timing may be associated with ethnicity, genetic, nutrition, coexisting disorders, medications, physical activity, and socioeconomic status [9]. However, there is limited knowledge about the association between small fetuses (SF) and early puberty timing in children. Previous studies have indicated that restricted prenatal growth may cause permanent alteration of the endocrine axes which can influence pubertal development. Birth weight is a major indicator to estimate the growth during fetal life. Intrauterine growth restriction (IUGR), small for gestational age (SGA) and low birth weight (LBW) are common manifestations of SF. IUGR generally should be assigned only to those infants with birth weight below the 10th percentile for gestational age with a pathologic restriction of fetal growth [10]. SGA is defined as neonates with a birth weight below a well-determined cut-off (10th, 5th, 3rd percentile) compared with the standards of a normal population. [11,12], while LBW refers to birth weight less than 2500 g [13]. 

To date, a number of studies have explored the association between puberty development and prenatal growth, however, they have shown inconsistent results. Hernández et al. [14] reviewed animal studies which correlated IUGR and changes in pubertal timing and found that rats or male lambs with IUGR had delayed pubertal development, while female lambs with IUGR had similar puberty timing as controls. Several population-based studies [15,16] found that the mean age at onset of puberty did not differ between the SGA group and the AGA (Appropriate for Gestational Age) group. By contrast, other studies [17,18] showed that SGA children started puberty significantly earlier than AGA children, and also showed an earlier age of menarche in girls who had fetal growth restriction compared to girls born of appropriate birth weight. The purpose of this study was to conduct a systematic review and meta-analysis to synthesize current scientific evidence and identify the association between SF and puberty timing in girls and boys.

## 2. Methods

### 2.1. Selection Criteria

The inclusion criteria included: (1) participants: children whose developmental stage was between pre-puberty and maturation; (2) exposure group consisted of IUGR, SGA, and LBW; (3) control group refers to those whose birth weights are between 2500 g and 4000 g or birth weights appropriate for gestational age; (4) main outcome measures were the number of pubertal children and age at pubertal events; (5) type of study design: only cohort studies were identified.

We excluded studies if (1) the subjects had other medical conditions or diseases that would cause early puberty or precocious puberty, such as intracranial tumor, adrenal cortical hyperplasia or tumors, gonadal tumor, thyroid hypofunction; (2) the outcome measures of interest were not reported.

### 2.2. Search Strategy

The databases including PubMed (1978 to February 2016), OVID (1946 to February 2016), Web of Science (1970 to February 2016), EBSCO (1976 to February 2016), CNKI (1979 to February 2016), WANFANG DATA (1987 to February 2016), CBM (1978 to February 2016), and CQVIP (1989 to February 2016) were searched using both the MeSH terms and free terms “fetal growth restriction” or “intrauterine growth retardation” or “low birth weight” or “small for gestational age”, in combination with “puberty” or “pubertal timing” or “puberty timing” or “sexual precocity” or “precocious puberty” or “premature pubarche” or “premature thelarche” or “menarche” or “first spermatorrhea” or “testicular volume” or “genitalia” or “pubic hair” or “armpit hair” or “beard” or “breast”. We modified the search strategy when searching in different databases. No language restrictions or restrictions on publication type were applied. All the retrieved literatures were entered into reference-managing software (EndNote, version X6, Thomson Scientific, Stamford, CT, USA).

### 2.3. Data Screening and Extraction

Two reviewers (Xu Deng, Wenyan Li) independently screened all the retrieved literature by titles and abstracts. The potential eligible studies were then screened again by full texts. The pre-designed criteria mentioned above were used to guide the entire process of screening. Subsequently, the following data were extracted from all the included studies using a pre-designed extraction form by two reviewers (Xu Deng, Wenyan Li): (1) general information, including authors, publication year, country; (2) study design and methodology quality; (3) follow up time; (4) participants characteristics and sample size; (5) the mark of onset of puberty; (6) outcome measures, including age at menarche (measured in years), age at onset of puberty (measured in years), number of pubertal children. Disagreements during screening and data extraction were resolved by discussion or consultation with the third reviewer (QL) to reach a consensus.

### 2.4. Risk of Bias Assessment

Two reviewers (Xu Deng, Wenyan Li) independently assessed the methodology quality of included cohort studies using the Newcastle–Ottawa Scale (NOS) (range, 0 to 9 scores) [19]. Assessment items included the selection (four items, four scores), comparability (one item, two scores), and assessment of outcome (three items, three scores). A final score >6 was regarded as high quality [20].

### 2.5. Statistical Analysis

Data analysis was performed using Review Manager Software (Version5.3, Cochrane Collaboration, London, UK). The continuous outcomes and dichotomous outcomes were assessed as weighted mean difference (WMD) and relative risk (RR) with 95% confidence intervals (CI), respectively. Results reported with median and ranges were converted to means and standard deviations according to Hozo et al. [21]. The statistical heterogeneity of the included studies was assessed by the χ^2^ test and I^2^ index. A random effects model was used when heterogeneity was found to be significant (I^2^ > 50% or *p* < 0.05); Otherwise, the fixed effects model was used. Subgroup analysis was conducted according to participants’ characteristics including gender and girls with or without precocious pubarche. Sensitivity analyses were conducted using the leave-one-out approach for all the outcomes. Publication bias was not assessed statistically due to the limited number of included studies for each outcome [22].

## 3. Results

### 3.1. Screening Results

**Figure 1:**
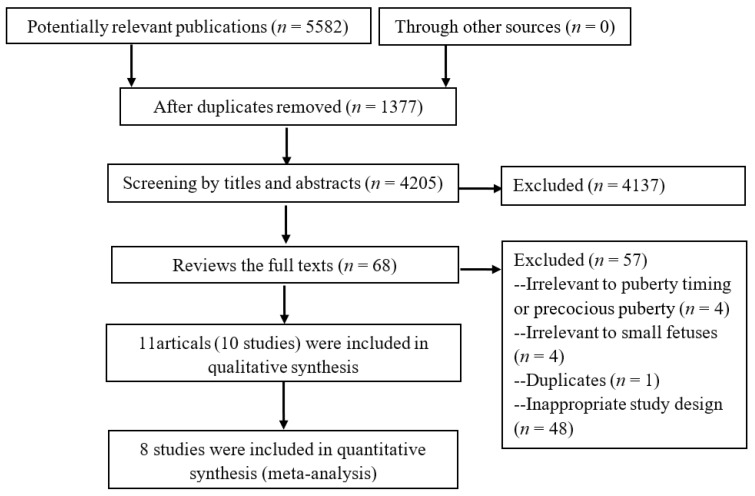
Flow diagram of literature search.

The process of study selection is shown in Figure 1 with an adapted PRISMA (Preferred reporting items for systematic reviews and meta-analyses) flow diagram [23]. A total of 5582 records were identified after searching the literature. After duplicate checking, 4137 out of 4205 studies were excluded after the initial screening of titles and abstracts. Ten cohort studies described in 11 articles [24,25,26,27,28,29,30,31,32,33,34] were included in the final review after a strict screening process based on eligibility criteria, of which, eight studies [24,25,26,27,28,29,30,31,32] involving 1399 subjects were included in the meta-analysis. The other 57 studies were excluded because they were not relevant to puberty timing or precocious puberty, or not relevant to small fetuses, or duplicate publication or not a cohort study. 

### 3.2. Characteristics of Included Studies

Table 1 presents the main characteristics of included studies. All ten cohort studies were published between 1982 and 2011, with a total number of 2366 subjects. Eight of the studies were conducted in higher income countries, and two studies were in a lower income country (India). Of the ten studies, five were with children who were LBW, and five were children with SGA, no studies enrolled children with IUGR. Definitions of LBW and SGA differed throughout studies. Five studies [27,28,30,31,32] defined SF by standard deviation (SD), including birth weight below −2 SD or below −1.5 SD; Three studies [24,33,34] defined SF by below 1500 g or 2000 g; One study [25,26] used birth weight below the 10th percentile corrected for gestational age, gender, and parity to define SF; One study [29] defined it used the India criterion. Only two studies [25,26,29] used the national reference standard to divide the SF group and control group. Although pubertal stages in most studies were assessed using the method of Marshall and Tanner, the criteria for defining onset of puberty varied across studies. Five studies [25,26,29,31,32,34] defined the onset of puberty by Tanner stage 2, including Tanner stage 2 of breast development (B2) for girls and Tanner stage 2 of genitalia development (G2) for boys; Two studies [28,30] used the time of growth spurt to define onset of puberty; One study [24] defined the onset of puberty as Tanner stage 3 of breast development (B3) in girls and testicular volume over 12 milliliters in boys; One study [27] defined it as with or without pubarche, and one study [33] did not specify the criteria of onset of puberty. The indicators evaluated in this study included number of pubertal children, age at onset of puberty, and age at menarche, which were estimated according to the mark of onset of puberty. At the end of follow-up, five studies [27,28,31,32,33] reported all the children occurred the main outcome; Two studies [24,30] reported the proportion who entered puberty was 80.6% and 62.4%; One study [25,26] reported 72.9% of children entered puberty at first follow-up and all children occurred the main outcome at second follow-up; One study [29] reported the proportion who entered puberty and menarche was 74.0% and 100.0%, respectively; One study [34] did not mention the proportion. Only eight studies were included in the meta-analysis, while the other two [33,34] were simply described because they only reported median, but the mean and standard deviation could not be calculated. Among the eight studies, seven studies assessed the age at menarche, three assessed the age at onset of puberty, and three reported the number of pubertal children.

### 3.3. Risk of Bias in Included Studies

Table 2 showed that eight studies were assessed as low risk of bias (scores of 7 and 8), while the other two were considered as moderate risk of bias (scores of 6). Two studies only included girls with precocious puberty as participants. All the studies were matched by age for both the exposed group and control group, except for one study which did not mention the measures used to control for confounding factors. For follow-up outcomes, three studies had follow-up rates less than 80% which were defined as inadequate follow up. The follow-up rate in one study [24] was 70% in the control group, while in other two studies the follow-up rates were 25% and 16% [33,34].

### 3.4. Meta-Analysis

#### 3.4.1. Number of Pubertal Children

**Figure 2:**
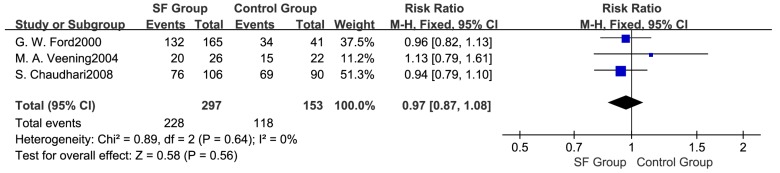
Forest plot for the number of pubertal children between SF group and Control group.

**Figure 3:**
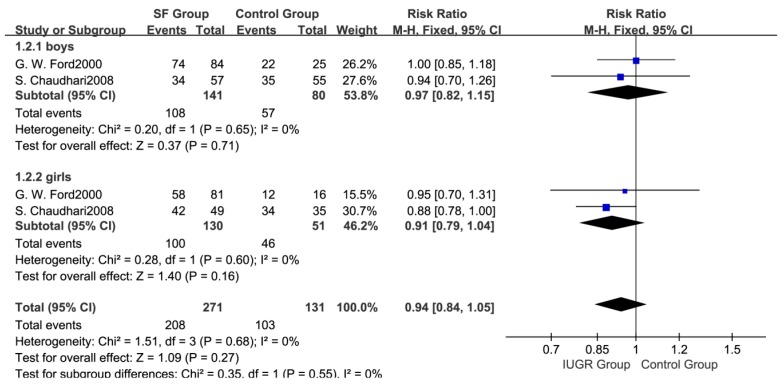
Forest plot for the number of pubertal children in boys and girls between SF group and Control group.

**Figure 4:**
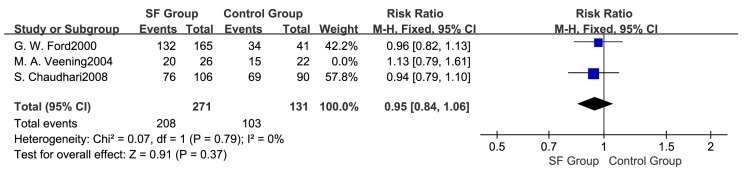
Sensitivity analysis for the number of pubertal children.

Meta-analysis based on three studies [24,25,29] indicated that there were no statistical differences in the total number of pubertal children between SF and control groups (RR: 0.97; 95% CI: 0.87 to 1.08, 450 subjects) (Figure 2). A fixed effects model was adopted (*p* > 0.05, I^2^ = 0%). Subgroup analysis (Figure 3) showed that there were no significant differences in the number of pubertal children either in boys (RR: 0.97; 95% CI: 0.82 to 1.15, 221 subjects) or girls (RR: 0.91; 95% CI: 0.79 to 1.04, 181 subjects) between two groups. In addition, the pooled results were not changed in each individual sensitivity analysis by the leaving one out approach (Figure 4).

#### 3.4.2. Age at Onset of Puberty

**Figure 5:**
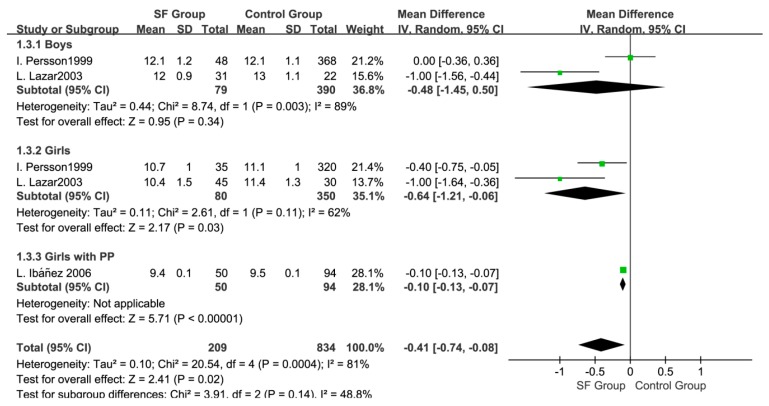
Forest plot for the age at onset of puberty between SF group and Control group.

Three studies [27,28,32] pooled the data of age at onset of puberty, showing that the onset of puberty in the SF group was earlier than that in the control group (WMD: −0.41; 95% CI: −0.74 to −0.08, 1043 subjects, Figure 5). The random effects model was adopted because of the high heterogeneity (*p* < 0.05, I^2^ = 81%). In subgroup analysis by gender, the SF group had an earlier onset of puberty than the control group in girls (WMD: −0.64; 95% CI: −1.21 to −0.06, 430 subjects), as did the pubarche (PP) girls with SF (WMD: −0.10; 95% CI: −0.13 to −0.07, 144 subjects). The results in boys indicated there were no statistical differences in age at onset of puberty between the two groups (WMD: −0.48; 95% CI: −1.45 to 0.50, 469 subjects).

One study [34] only reported the median age at onset of puberty for boys and girls, but the standard deviation could not be calculated based on the reported data. The study reported that the median age for genitalia stage 2 was similar in the boys who had LBW (10.2 years) and controls (10.02 years). However, the median age for breast stage 2 in girls from the LBW group was 10.7 years compared to 11.1 years in the controls.

#### 3.4.3. Age at Menarche

**Figure 6:**
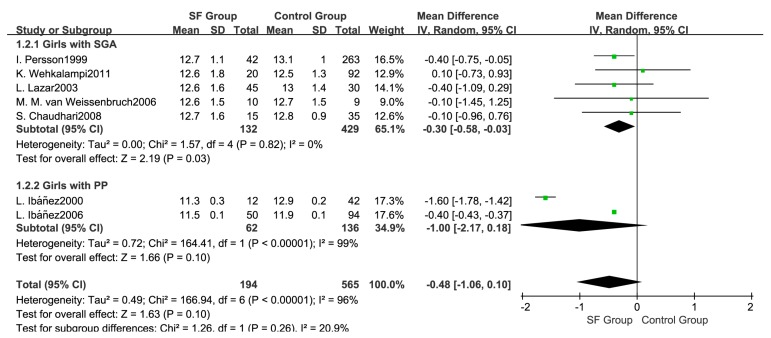
Forest plot for the age at menarche between SF group and Control group.

Data on age at menarche was provided in seven studies [26,27,28,29,30,31,32]. The pooled data showed that SF was not associated with early age at menarche in girls (WMD: −0.48; 95% CI: −1.06 to 0.10, 759 subjects. (Figure 6). The random effects model was adopted (*p* < 0.05, I^2^ = 96%). Subgroup analysis showed that girls with SGA had an earlier age at menarche than controls (WMD: −0.30; 95% CI: −0.58 to −0.03, 561 subjects). In girls with PP, both studies showed an earlier age at menarche in the SF group than in the control group, but the pooled estimate showed the ages at menarche were not different between two groups (WMD: −1.00; 95% CI: −2.17 to 0.18, 198 subjects).

**Figure 7:**
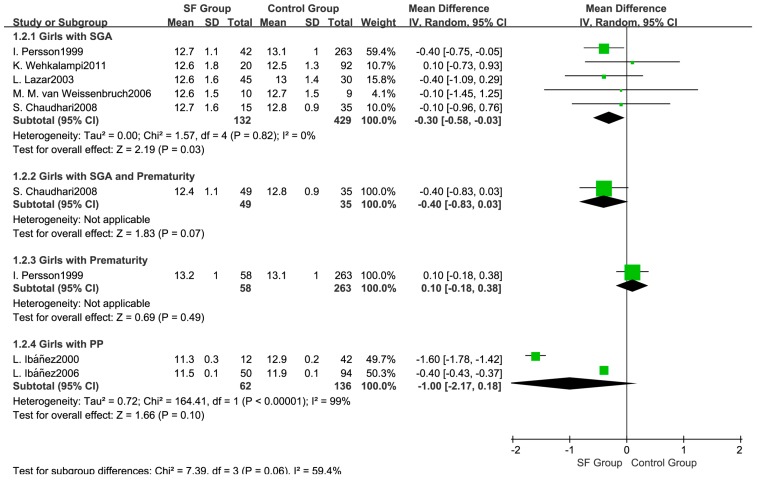
Forest plot for subgroup analysis on age at menarche between SF group and Control group.

**Figure 8:**
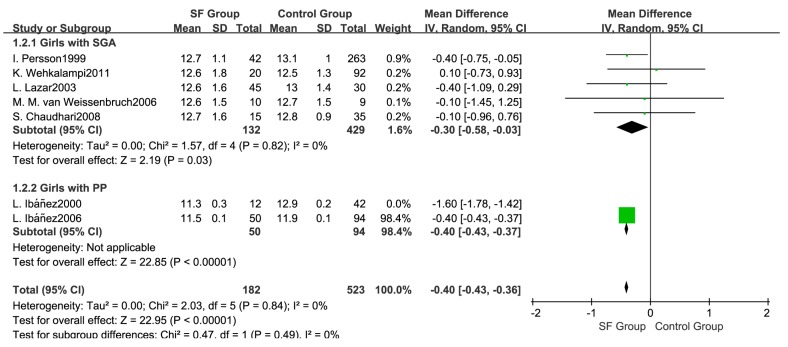
Sensitivity analysis for the age at menarche.

Considering the influence of prematurity, another subgroup analysis was conducted (Figure 7). Age at menarche between the SF group and the control group was not significantly different in either the prematurity subgroup (WMD: 0.10; 95% CI: −0.18 to 0.38, 321 subjects) or in the SGA with prematurity subgroup (WMD: −0.40; 95% CI: −0.83 to 0.03, 84 subjects). After removing the study by Ibáñez et al. [31] in the sensitivity analysis, the pooled results indicated an earlier age at menarche in the SF group (WMD: −0.40; 95% CI: −0.43 to −0.37, 705 subjects), and the heterogeneity was reduced to 0% (Figure 8).

Two studies [33,34] which only reported the mean or median age at menarche without standard deviation could not be included in the meta-analysis. In the Fledelius et al. study [33], the mean age at menarche in LBW girls was 13.5 years, which was 0.5 years later than full-term girls. Bhargava et al. [34] reported that menarche occurred at a median age of 13.6 years in the controls, which occurred 6 months earlier in the preterm girls and 12 months earlier in LBW girls.

## 4. Discussion

The initiation and progression of puberty is complex and many factors have been identified that either directly or indirectly influence the hypothalamic-pituitary-gonadal axis. With the increase of gonadotropin-releasing hormone (GnRH) secretion, the luteinizing hormone (LH) and the follicle-stimulating hormone (FSH) secretion by pituitary also increased [35]. LBW has been associated with higher levels of FSH and LH, lower levels of inhibin-B and testosterone, smaller testicular volume in adolescence, and subfertility [36]. As an adrenal androgen, dehydroepiandrosteronesulfate (DHEAS) levels were increased in children with lower birthweight [13,17]. The pathophysiological mechanism underlying the pubertal growth with children born SF remains unclear, but population studies have provided more evidence that SF is associated with children’s onset of puberty.

This meta-analysis found that compared with the control group, the SF group experienced onset of puberty about five months earlier in all children, of which, SF girls experienced onset of puberty about eight months earlier than normal girls. This indicates that SF may be associated with an earlier onset of puberty. Menarche occurred within the normal age range in both groups, but was significantly earlier (0.3 years earlier) in girls born with SGA. Some case-control studies have also found results consistent with these findings [37,38,39,40]. Previous studies suggest that SF is related to a higher risk for obesity, cardiovascular disease, and chronic metabolic diseases in adulthood [41,42], as well as in children with earlier onset of puberty and girls who have earlier menarche [43,44]. However, the underlying mechanisms still need further study. The study by Fledelius et al. 1982 [33] which could not be included in our meta-analysis, reported that the menarche in LBW girls appeared half a year later than in the full-term controls. The reason for this contrasting result may be that the weight of the LBW group in this study was below 2000 g, indicating that a very low birth weight is an obstacle to puberty development. 

Subgroup analysis was conducted to explore the potential confounding factors in this meta-analysis. We did not find any significant difference in age at menarche between the SF group and the control group in the prematurity subgroup. However, only two studies with limited samples provided the data of prematurity, which may not reflect the real association. Therefore, more related studies are still needed to identify potential effects of prematurity. 

We did not detect statistical differences in the number of pubertal children between the SF group and the control group, which may be due to different criteria of defining onset of puberty in the included studies. For example, Ford et al. [24] defined the onset of puberty as breast stage Tanner stage 3 and above in girls and testicular volume over 12 mL in boys, while the other two studies [25,29] used the criteria breast stage over Tanner stage 2 in girls and testicular volume over 4 mL and 8 mL in boys respectively. Further, the overall proportion of children entering puberty would be expected to differ by age. The oldest age at follow up for the three studies was 12, 14 or 15, while the proportion of pubertal was 72.9%, 74% and 80.6%, respectively. Stronger association between SF and pubertal development might be found if the final assessment of puberty was conducted at an earlier age. Moreover, it also might be the part of the reason that the results differ for age at puberty versus the number of pubertal children between the SF group and control group.

The heterogeneity of the pooled estimates decreased when conducting subgroup analysis by sex and by PP, which indicated that sex and PP in girls were the potential sources of heterogeneity. Regarding the age at onset of puberty, inconsistent results were found in two subgroups, which suggested that SF may have differential effects on pubertal development in boys and girls. The high heterogeneity may be due to the marked differences in sample sizes between the two included studies and the notable difference in the age at onset of puberty between SF and controls in the Lazar et al. study. As to age at menarche, when the results of Ibáñez et al. [31] are included in the meta-analysis, the differences in age at menarche between the SF and control groups was not significant. In fact, both the results of the two different studies by Ibáñez et al. [30,32] showed an earlier age at menarche in the SF group, but once the two results were pooled, the differences were not statistically significant with high heterogeneity. This is probably due to the lack of the upper limit for birth weight range of the control group in Ibáñez et al. [31], which means the controls included normal birth weight children, as well as large birth weight children. The large birth weight children may have a delayed puberty timing which enlarges the differences between the two groups. 

We conducted this study following the rigorous systematic review and meta-analysis approach recommended by the Cochrane Handbook for Systematic Reviews and the PRISMA statement [23,45]. A review protocol was developed beforehand to guide the process of review and meta-analysis. The included studies were relatively high quality cohort studies based on NOS assessment. However, several limitations still need to be considered when interpreting and generalizing the present results. First, not all the eligible studies were included in meta-analysis due to inappropriate comparisons or lack of necessary data reported in the original studies, such as standard deviation for age at onset of puberty and number of pubertal children in both groups. A large Hong Kong study was excluded because the outcome measures of interest were not reported or calculated [46]. In this study, the author indicated that SGA was not associated with earlier puberty after adjustment for other variables. A study by Sorensen et al. [47] found that earlier age at menarche was associated with lower birth weight in twin girls. This study was excluded from our meta-analysis as it did not compare age at menarche between the LBW/SGA group and control group. Second, subgroup analyses were only conducted by gender and girls with or without PP in this study due to the limited number of included studies. Heterogeneity that comes from regional diversity, ethnicity diversity, nutritional intake differences, sample size differences, and health status could not be analyzed in the current meta-analysis, which needs to be considered when interpreting the pooled results. Third, the diversity of definitions of both SF and puberty could lead to a bias in the data analysis. Fourth, people with PP seem to be a truncated population that is unlikely to provide fruitful information about the impact of SF on puberty. Fifth, the age at outcome may be abnormal distribution as not all of the children have achieved the outcome, and a mean value to measure the central tendency could also lead to a bias in the meta-analysis. Further, most included studies had small sample sizes. Analyses of epidemiological studies have demonstrated that studies with a sample size of less than 100 would increase the type I error [48]. Moreover, studies were mainly conducted in higher income countries, which might limit the generalizability of the results.

## 5. Conclusions

As far as we know, the present study is the first attempt to determine the association between SF and puberty timing by a systematic review and meta-analysis. From the pooled results of individual studies, our findings suggest that SF may be associated with earlier age at onset of puberty, especially among girls, as well as earlier age at menarche for girls. Nevertheless, as the evidence of this study was mostly from higher income countries, well-designed cohort studies with large sample sizes and long-term follow-up among different countries and ethnicities are needed.

## Figures and Tables

**Table 1:** Characteristics of studies included in the systematic review.

Study	Sample Size, *n* SF/Control	Age Range	Follow-Up Period	Type of SF	Definition of SF	Reference Standard	Country/Areas	Main Outcomes	Mark of Onset of Puberty	Proportion of Individuals Entered Puberty, %
Ford 2000 [24]	206/60	0–14 years	1977–1996	LBW	Birth weight <1500 g	This study	Australia	No. of breast stage > 3, No. of testicular volume ≥ 12 mL	B3 for girls, Testicular volume > 12 mL for boys	80.6
Veening 2004 [25]	29/24	9–15 years	1980–1983	SGA	Birth weight below the 10th percentile corrected for gestational age, gender and parity	Dutch reference	Netherlands	No. of breast stage > 2, No. of testicular volume ≥ 4 mL, Age at menarche	B2 for girls, Testicular volume > 4 mL for boys	72.9
Weissenbruch 2006 [26]										100.0 *
Lazar 2003 [27]	76/52	0–14 years	Not mentioned	SGA	Birth weight <−2 SD for gestational age	This study	Israel	Age at menarche, age at onset of puberty	Pubertal signs (gonadarche with or without pubarche) appeared	100.0 *
Persson 1999 [28]	101/806	0–18 years	1973–1995	SGA	Birth weight <−2 SD for gestational age	This study	Sweden	Age at onset of puberty, age at menarche	Growth spurt	100.0 *
Chaudhari 2008 [29]	73/90	0–15 years	1987–2001	SGA	India criterion	India reference	India	No. of Tanner stage > 2, age at menarche	B2 for girls, G2 for boys	74.0, 100.0 *
Wehkalampi 2011 [30]	35/146	2–16 years	1978–1985	SGA	Birth weight <−2 SD	This study	Finland	Age at menarche	Growth spurt	62.4 *
Ibáñez 2000 [31]	12/42	Not mentioned	Not mentioned	LBW	Birth weight <−1.5 SD	This study	Spain	Age at menarche	B2 for girls	100.0 *
Ibáñez 2006 [32]	50/94	Not mentioned	Not mentioned	LBW	Birth weight <−2 SD	This study	Spain	Age at onset of puberty, Age at menarche	B2 for girls	100.0 *
Fledelius 1982 [33]	302/237	10–18 years	1959–1979	LBW	Birth weight <2000 g	This study	Denmark	Age at menarche	Not mentioned	100.0 *
Bhargava 1995 [34]	252/176	0–14 years	1968–1985	LBW	Birth weight <2000 g	This study	India	Age at menarche	B2 for girls, G2 for boys	Not mentioned

SF—small fetuses; SGA—small for gestational age; LBW—low birth weigh; SD—standard deviation; B—breast development stage; G—genitalia development stage. * proportion of individuals occurred menarche.

**Table 2:** The Newcastle–Ottawa Scale (NOS) for assessing the methodology quality.

Study	Selection				Comparability	Outcome			Quality Scores
	Representativeness of the Exposed Cohort	Selection of the Non Exposed Cohort	Ascertainment of Exposure	Outcome of Interest Was Not Present at Start of Study	Comparability of Cohorts on the Basis of the Design or Analysis	Assessment of Outcome	Was Follow-Up Long Enough for Outcomes to Occur	Adequacy of Follow Up of Cohorts	
Ford2000 [24]	1	1	1	1	1	0	1	0	6
Veening 2004 [25]	1	1	1	1	1	1	1	1	8
Weissenbruch 2006 [26]									
Lazar 2003 [27]	1	1	1	1	1	1	1	1	8
Fledelius 1982 [33]	1	1	1	1	1	1	1	0	7
Persson 1999 [28]	1	1	1	1	1	1	1	1	8
Chaudhari 2008 [29]	1	1	1	1	1	1	1	1	8
Bhargava 1995 [34]	1	1	1	1	1	1	1	0	7
Ibáñez 2000 [31]	0	1	1	1	0	1	1	1	6
Wehkalampi 2011 [30]	1	1	1	1	1	1	1	1	8
Ibáñez 2006 [32]	0	1	1	1	1	1	1	1	7
